# Supplementary material for: Predicting visual function by interpreting a neuronal wiring diagram
Source: Nature. 2024 Oct 2;634(8032):113–23. doi: 10.1038/s41586-024-07953-5 (PMC11446822; doi:10.1038/s41586-024-07953-5)
Supplement: Supplementary file 6 — CRF and ERF predictions for individual TmY4 and TmY9 cells. Analogous to Supplementary Data 3, but for TmY target types. Shown are the top four monosynaptic pathways, the strongest pathway passing through each of the top ten intermediary types (ranking from Extended Data Fig. 7), and the trisynaptic pathway Tm1–TmY–Dm3–TmY (see the section entitled Prediction of spatial normalization). [file 41586_2024_7953_MOESM6_ESM.zip › DataS4/TmY9q/720575940620947638.pdf]

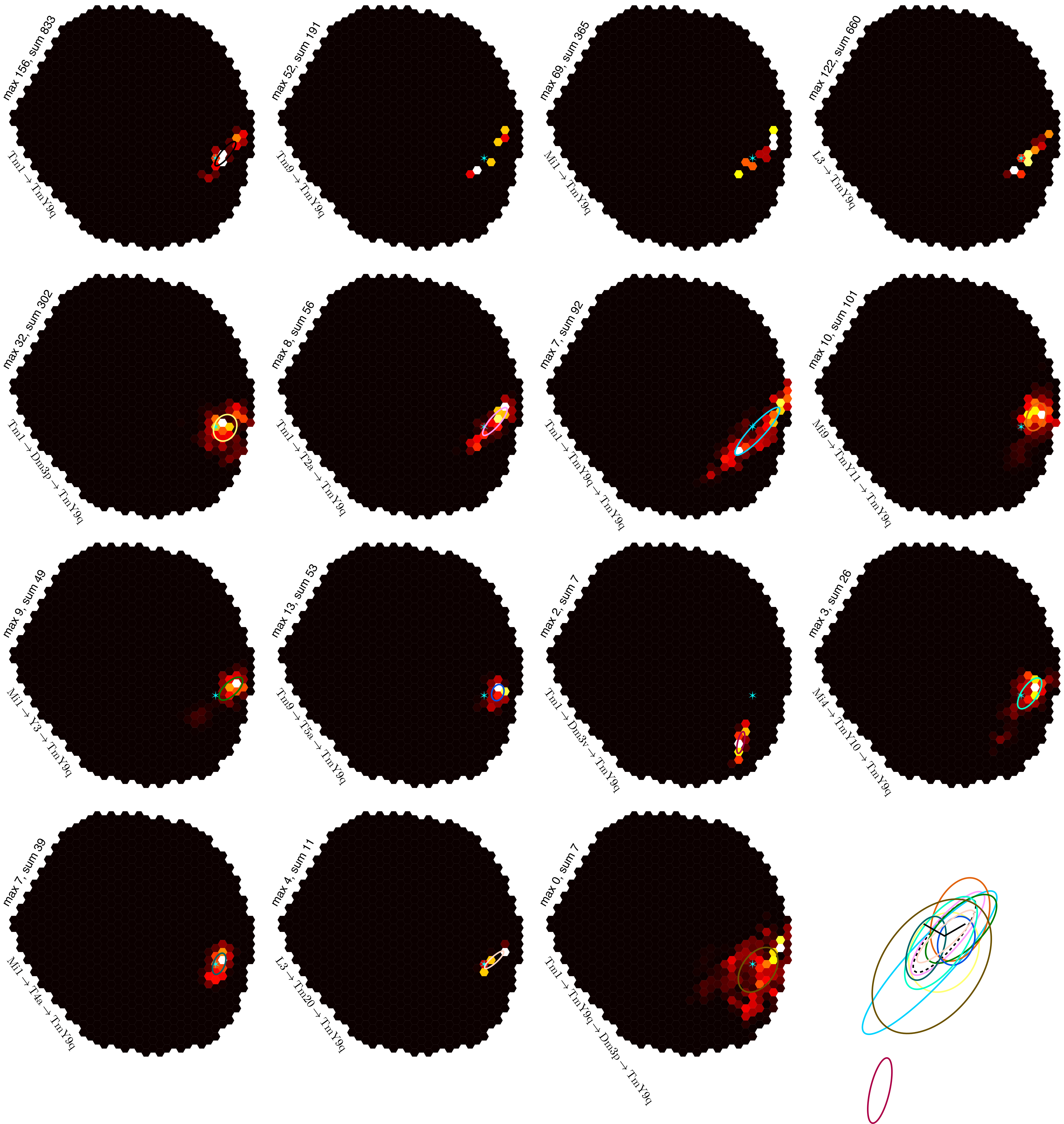
max 156, sum 833
Tm1 → TmY9q
max 52, sum 191
Tm9 → TmY9q
max 69, sum 365
Mi1 → TmY9q
max 122, sum 660
L3 → TmY9q
max 32, sum 302
Tm1 → Dm3p → TmY9q
max 8, sum 56
Tm1 → T2a → TmY9q
max 7, sum 92
Tm1 → TmY9q → TmY9q
max 10, sum 101
Mi9 → TmY11 → TmY9q
max 9, sum 49
Mi1 → Y3 → TmY9q
max 13, sum 53
Tm9 → T5a → TmY9q
max 2, sum 7
Tm1 → Dm3v → TmY9q
max 3, sum 26
Mi4 → TmY10 → TmY9q
max 7, sum 39
Mi1 → T4a → TmY9q
max 4, sum 11
L3 → Tm20 → TmY9q
max 0, sum 7
Tm1 → TmY9q → Dm3p → TmY9q
